# Production, recovery, and purification of recombinant 503 antigen of *Leishmania infantum chagasi *using expanded bed adsorption chromatography

**DOI:** 10.1186/1753-6561-8-S4-P194

**Published:** 2014-10-01

**Authors:** Francisco Canindé de Sousa Junior, Michelle Rossana Ferreira Vaz, Carlos Eduardo de Araújo Padilha, Daniella Regina Arantes Martins, Gorete Ribeiro de Macedo, Everaldo Silvino dos Santos

**Affiliations:** 1 Departamento de Engenharia Química, Universidade Federal do Rio Grande do Norte, Natal, RN, 59078-970, Brazil; 2 Centro de Desenvolvimento Sustentável do Semiárido, Universidade Federal de Campina Grande, Sumé, PB, 58540-000, Brazil; 3 Departamento de Biologia Celular e Genética, Universidade Federal do Rio Grande do Norte, Natal, RN, 59078-970, Brazil

## Background

Visceral leishmaniasis, a disease caused by *Leishmania infantum chagasi*, represents a major public health problem in many areas of the world. Despite the considerable effort, there is no effective and safe vaccine for human use [1]. Some authors have reported that as much as 50% of overall costs in the biotechnology industries are related to downstream processing. Thus, the development of new and economically advantageous purification methods is a challenge [2]. Expanded bed adsorption (EBA) is an innovative chromatography technology that allows the adsorption of target proteins directly from unclarified feedstock. EBA technology combines solid-liquid separation with an adsorption step in a single-unit operation, aiming at increased overall yield, reduced operational time, and less capital investment and consumables [3,4]. Thus, the aim of this work was to purify the 503 antigen of *Leishmania i. chagasi *directly from crude feedstock using EBA chromatography.

## Methods

The strain of *E. coli *expressing 503 antigen of *Leishmania i. chagasi *was kindly provided by Dr. Mary Wilson (University of Iowa, USA) [1]. The clone was cultured in 2xTY medium supplemented with antibiotics [5]. The cultivations were carried out using a bench bioreactor with a work volume of 1.5 L, at frequency of agitation of 400 rpm and constant output aeration of 1 vvm. The expression of the recombinant protein was induced by the addition of lactose 10 g/L. Optimization of adsorption and elution conditions of 503 antigen was performed in batch mode according to two central composite designs. Then, EBA using Streamline Chelating was employed to purify 503 antigen from unclarified bacterial homogenate with a glass column (30.0 cm × 2.6 cm I.D) and an adjustable piston, in order to minimize headspace over the fluidized bed. Analysis of the fractions was performed by Lowry method and electrophoresis on 15% polyacrylamide gels under denaturing conditions. The gels were photographed to estimate protein production, using the software ImageJ.

## Results and conclusions

The batch adsorption experiment with Streamline Chelating showed that the optimal binding condition of 503 antigen was pH 8.0 in the presence of 1.625M NaCl. The optimal elution condition for the elution of protein of interest from the adsorbent was in the presence of 600mM imidazole. The adsorption isothermal data of 503 antigen onto Streamline Chelating showed that the data obeyed the Langmuir adsorption isotherm. The EBA assays showed that bed height increased linearly with the linear flow velocity. The fraction recovered after the elution contained 25% of the initial amount of 503 antigen. In conclusion, EBA has been applied successfully to purify the 503 antigen from an *E. coli *homogenate. The EBA mode combined clarification, capture, and purification of the interesting protein in a single step process, thereby giving rise to a good product recovery.
